# Characterizing the expression of the human olfactory receptor gene family using a novel DNA microarray

**DOI:** 10.1186/gb-2007-8-5-r86

**Published:** 2007-05-17

**Authors:** Xiaohong Zhang, Omar De la Cruz, Jayant M Pinto, Dan Nicolae, Stuart Firestein, Yoav Gilad

**Affiliations:** 1 Department of Biological Sciences, Columbia University, New York, NY 10027, USA; 2 Department of Statistics, University of Chicago, Chicago, IL 60637, USA; 3 Department of Human Genetics, University of Chicago, Chicago, IL 60637, USA; 4 Department of Medicine, University of Chicago, Chicago, IL 60637, USA

## Abstract

**Background:**

Olfactory receptor (OR) genes were discovered more than a decade ago, when Buck and Axel observed that, in rats, certain G-protein coupled receptors are expressed exclusively in the olfactory epithelium. Subsequently, protein sequence similarity was used to identify entire OR gene repertoires of a number of mammalian species, but only in mouse were these predictions followed up by expression studies in olfactory epithelium. To rectify this, we have developed a DNA microarray that contains probes for most predicted human OR loci and used that array to examine OR gene expression profiles in olfactory epithelium tissues from three individuals.

**Results:**

We detected expression of 437 (76%) human OR genes in these olfactory epithelia. Interestingly, we detected widespread expression of OR pseudogenes, an observation that may shed light on the mechanism of OR gene choice in the olfactory sensory neurons. To address the hypothesis that OR genes may carry out additional functions, we also characterized the expression of OR genes in a number of non-olfactory tissues.

**Conclusion:**

While our results corroborate the functional annotation of the majority of predicted human odorant receptors, we find that a large number of putative human OR genes are expressed in non-olfactory tissues, sometimes exclusively so. Our evolutionary analysis of ectopically expressed human OR genes does not lend support to the hypothesis that these genes have alternative functions.

## Abstract 2

Using a microarray, expression of 76% of predicted human olfactory receptor genes was detected in olfactory epithelia, and many were expressed in non-olfactory tissues.

## Background

Buck and Axel [1] identified the odorant receptor (OR) gene family based partly on the observation that OR genes were expressed in olfactory epithelium, but were not detected in lung, liver, spleen, kidney, retina, and brain. Subsequently, additional OR genes were recognized in genomic sequences by their similarity to the first set of identified OR genes [2,3], and by the presence of certain predicted protein motifs [1,4]. Recently, the complete genomic sequence of a number of mammalian species became available, permitting interspecies comparisons of complete OR gene repertoires. The analyses of published mammalian genomes suggested that the original estimate of the size of the mammalian OR gene repertoire, approximately 100 genes, was a severe underestimate. Indeed, it is now thought that mammalian genomes carry 800-1,400 OR genes [5-10], which are typically organized in gene clusters and are found on many chromosomes. With roughly 3% of all genes coding for odorant receptors, OR genes are by far the largest gene family in mammalian genomes.

To date, however, mammalian OR genes have remained largely orphan receptors. In fact, until recently, there was no systematic study of putative mammalian OR gene expression in olfactory epithelium [7,11], such that the functional annotation of OR genes remained unclear. Moreover, expression of several predicted mammalian OR genes was detected only in non-olfactory tissues, notably in testis [12-14]. These observations raised the possibility that a subset of predicted OR genes may not be odorant receptors at all but have other functions, with important implications for functional studies in olfaction and comparisons of mammalian OR gene repertoires. Alternatively, OR genes may have a function beyond odor recognition, for example, in sperm chemotaxis [15].

Recently, Zhang *et al*. [16] studied the expression of nearly all predicted OR genes in mouse using a newly developed DNA microarray [16]. Most (approximately 80%) predicted mouse OR genes were confirmed to be expressed in olfactory epithelium, but a subset were found to be expressed only in non-olfactory tissues and, consequently, their functional annotation is now in question [16]. In humans, it is not known how many of the predicted OR genes are expressed in the olfactory epithelium, and hence how many are likely to participate in odorant binding. Moreover, the predicted human OR gene repertoire includes nearly 600 pseudogenes [5] and it remains unknown how often they are expressed. Since olfactory sensory neurons are believed to express only a single functional OR gene, if these pseudogenes are routinely expressed in the olfactory epithelium, a large proportion of neurons may either express a single non-functional gene, or co-express a functional and non-functional OR genes [17].

A recent study [14] used expressed sequence tag (EST) data and results of genome-wide microarrays to survey human OR gene expression in olfactory epithelium and several non-olfactory tissues. However, that analysis was limited by shortcomings of the available data, including biases and inaccuracies in the EST databases and incomplete sampling of OR genes on the human genome-wide microarray (which includes probe-sets for only 356 predicted OR genes and pseudogenes). Moreover, the genome-wide microarray was not optimized specifically to measure OR gene expression, so many of the probes may be susceptible to cross-hybridization by other OR genes [14]. Indeed, the authors' analysis of the probe-set sequences suggested that the expression of only 217 human OR genes and pseudogenes could be estimated with confidence using the genome-wide microarray data [14].

To comprehensively and reliably assess expression of predicted human OR genes, we designed a new microarray with probes for nearly all human OR genes. We used this microarray to characterize the expression of human OR genes in olfactory epithelium as well as in a number of other tissues.

## Results and discussion

**Figure 1:**
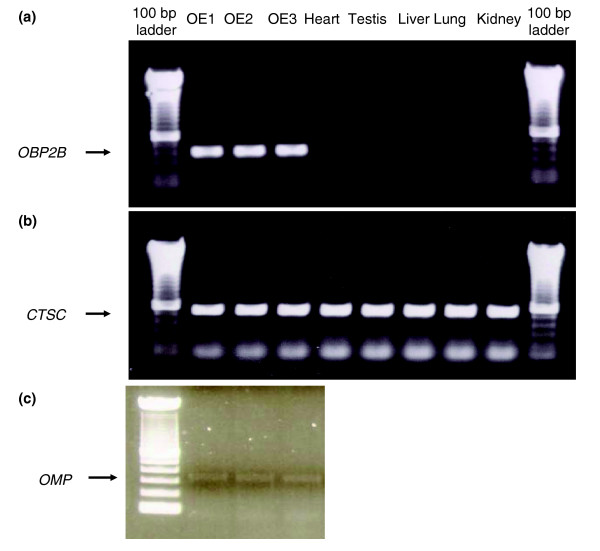
Gel electrophoresis of PCR amplification results using cDNA from three olfactory epithelium tissues, heart, testis, liver, lung, and kidney as template. **(a) **The 440 base-pair (bp) *OBP2B *product was only amplified from the olfactory epithelium samples. **(b) **The 562 bp cathepsin C (*CTSC*) product was successfully amplified from all three samples. Both primer pairs were designed to amplify multiple exon products and hence are expected to yield a much larger product (1,686 bp) if genomic DNA was used as template. **(c) **The 378 bp product of the *OMP *gene was amplified from the olfactory epithelium samples to confirm that these samples contain neutrons.

To measure the expression of human OR genes, we extracted total RNA from three samples of human olfactory epithelium tissues collected by the National Disease Research Interchange)[18] within eight hours of the donor's death. We confirmed that RNA was extracted from olfactory epithelium tissues by amplification of the odorant binding protein 2B (*OBP2B*) gene (Figure 1), which is expressed exclusively in olfactory epithelium [19]. In addition, we tested for the presence of olfactory sensory neurons in each sample by amplifying the olfactory sensory neuron marker gene, the olfactory marker protein (*OMP*) [20]. Once we confirmed the source of the RNA, we proceeded by labeling and hybridizing each olfactory epithelium RNA sample, in two independent technical replicates, to a custom human OR gene microarray (see Materials and methods). Similarly, we hybridized RNA from human liver, lung, kidney, heart, and testis (purchased from Ambion (Austin, TX, USA)) to the microarray in two technical replicates each.

### Expression of OR genes in human olfactory epithelium

Our first goal was to detect which of the predicted human OR genes are expressed in olfactory epithelium, thereby lending support to their functional annotation as odorant receptors. One way to examine this is to rely on the absent/present calls that the Affymetrix software provides for each probe-set. However, microarrays were not designed to detect expression but rather to compare levels of expression between samples or treatments and, as a result, existing algorithms are not well suited for our application [21,22]. In particular, as probes vary in their specificity and sensitivity, using cut-offs for absolute hybridization intensity as a detection tool is unreliable [23-25]. An alternative is to use a comparison of gene expression levels across the studied RNA samples in order to detect genes that are expressed [16]. The rationale of this approach is that genes with significantly higher expression in sample A compared to sample B are clearly expressed in A. Thus, OR genes with significantly elevated expression in olfactory epithelium compared to other tissues can be considered 'detected'. Accordingly, we compared expression of OR genes in the olfactory epithelium samples with their expression levels in the five non-olfactory tissues (see Materials and methods).

**Figure 2:**
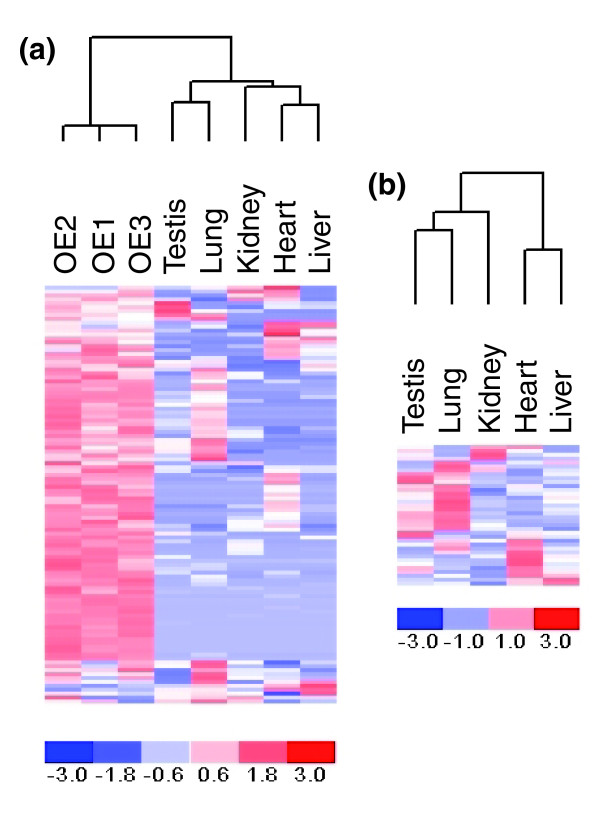
Expression profile of human OR genes across tissues. The log transformed detection *P *values for OR genes in all tissues (from Additional data file 1) were standardized to have mean 0 and standard deviation 1 and are color coded (red and blue shades indicate values above and below the mean, respectively). The dendrograms on top of each panel illustrate the clustering (by hierarchical clustering in dchip [48]) of tissue samples based on the profile of OR gene expression. **(a) **All 578 predicted OR genes are included in a comparison between olfactory epithelium (OE) and the non-olfactory tissues (see Materials and methods). **(b) **Shown are the data for only the 147 OR genes with significantly elevated expression in non-olfactory tissues.

We found 437 predicted OR genes on the array to be expressed in human olfactory epithelium at *P *< 0.05 (Figure 2a; Additional data file 1), when at most 29 are expected by chance, given the statistical cutoff and the number of genes on the array (see Table 1 for results using alternative statistical cutoffs). These results were validated by performing RT-PCR on ten randomly chosen OR genes whose expression was detected in olfactory epithelium (Additional data file 1); in all ten cases, the RT-PCR confirmed the arrays results. Thus, we confirmed that the vast majority (76%) of predicted human OR genes are indeed expressed in olfactory epithelium. In contrast, the functional annotations of 141 predicted human OR genes are now in question, as these were not detected as expressed in olfactory epithelium. We note, however, that 109 of the above 141 OR genes did not have significantly elevated expression levels in any tissue (Additional data file 1). Since our detection criterion is based on differential expression, we cannot exclude the possibility that a subset of the 109 OR genes were not detected because they are expressed at similar levels in all tissues, including olfactory epithelium [16].

**Table 1:** Number of expressed OR genes in human olfactory epithelium

*P *value*	No. of detected OR genes and pseudogenes	Only intact^†^	Only pseudogenes^†^
0.001	192 (33%)	131 (36%)	61 (29%)
0.01	342 (59%)	232 (63%)	110 (52%)
0.05	437 (76%)	295 (80%)	142 (67%)

*The statistical cutoff used to identify OR genes as expressed. ^†^The number and percentage (in parenthesis) of intact OR genes and pseudogenes on the array detected as expressed. The array includes probe-sets for 578 predicted human OR genes.

Previous studies also noted anecdotal expression of individual human OR pseudogenes [11,14]. Of the 212 human OR pseudogenes on the microarray, 142 (67%) were found to be expressed in olfactory epithelium (Additional data file 1). While considerable, this fraction is significantly lower than the fraction of intact OR genes, 295 out of 366 (80%), found to be expressed in olfactory epithelium (Fisher Exact test, *P *< 10^-3^; see Table 1 for results using alternative statistical cut-offs). Moreover, intact OR genes appear to be expressed at a higher level on average than OR pseudogenes (assuming that the probe effect is canceled in large samples, such that we can compare hybridization intensity across groups of probe-sets, the mean difference in normalized intensity is +16%; Mann-Whitney-U, *P *= 0.005). The more frequent and greater expression of genes relative to pseudogenes is consistent with recent observations in mice (XZ and SF, manuscript in preparation). The observation of widespread OR pseudogene expression in this study, as well as a number of others [11,14], suggests that a nonsense-mediated decay RNA system (reviewed in [26]) does not efficiently remove OR pseudogene mRNAs.

On our array, we included only OR pseudogenes with one or two premature coding region disruptions. These ORs are likely to be recent pseudogenes, so that expression in olfactory epithelium may not be unexpected. This said, the observation of widespread OR pseudogene expression has implications for the outstanding question of how OR gene expression is regulated in olfactory sensory neurons. A common model is one in which mature olfactory sensory neurons are assumed to have a cellular mechanism that restricts the expression to only one OR gene [27,28]. This model predicts that a neuron expressing a pseudogene would eventually switch to express a functional gene [27,28], while a functional and an OR pseudogene would rarely be co-expressed. Thus, if OR pseudogene expression is widespread, as our observations suggest, this implies that, at any given time, a large proportion of neurons will not express functional genes and, thus, will not contribute to the sense of smell. This prediction is consistent with the small numbers of OR pseudogenes found in species that rely heavily on their sense of smell [29].

An alternative model is that expression of OR pseudogenes in olfactory sensory neurons occurs with the same probability as expression of intact OR genes, but that the neurons expressing only a non-functional OR gene do not converge in the olfactory bulb, never reach maturation and are removed [17], while neurons that co-express both a functional gene as well as a pseudogene will converge in the olfactory bulb [17]. This model does not rely on a cellular mechanism that restricts the expression to only one OR gene. Accordingly, this model predicts that pseudogenes can be either co-expressed with a functional gene, or are limited to young neurons that are removed. This prediction is consistent with our observations of a lower proportion and weaker expression of OR pseudogenes compared to intact genes. If true, it would suggest that a considerable proportion of human olfactory sensory neuron cells co-express a functional as well as an OR pseudogene.

### Inter-individual variation in OR gene expression

**Figure 3:**
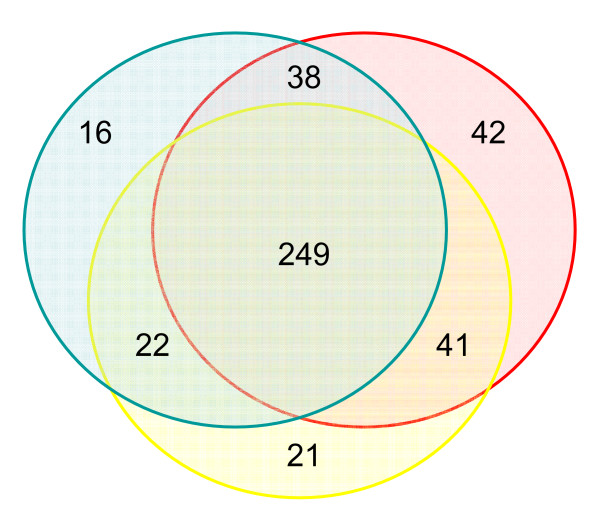
The number of predicted human OR genes whose expression was detected (at *P *< 0.05) in one or more of the three olfactory epithelium (OE) samples. As can be seen, there is a substantial difference in the expressed OR gene repertoire of each of the three OE samples.

Recent work suggested that the extensive genetic variation in human OR protein coding regions (in particular, segregating null mutations) may account for inter-individual variability in the sense of smell [30,31]. Here, we also find evidence that the repertoire of expressed OR genes varies across individuals (Figure 3). Although our sample of only three olfactory epithelium tissues is insufficient to quantify the variability in human OR gene expression, it suggests that such variability is abundant. Regardless of the statistical cutoff (10^-5 ^<*P *< 0.05) employed to identify OR genes that are expressed in each sample, the expressed OR gene repertoires of any pair of individuals differs by at least 14%. This number reflects technical error as well as true inter-individual differences. However, we do not observe significant differences in the expression of the 'house-keeping' genes across the olfactory epithelium tissues, indicating that technical explanations are unlikely to account for the difference in detection of OR gene expression across these samples. Thus, our findings raise the possibility that, in addition to differences in protein function, variation in the regulation of OR genes also underlies phenotypic differences in olfactory sensitivity between individuals. If so, studies of the genetic basis of specific anosmia should include genetic variants in OR gene promoter and putative control regions in addition to coding region polymorphisms.

### Expression of OR genes in non-olfactory epithelium tissues

We detected OR gene expression in five non-olfactory tissues by identifying genes whose expression is significantly elevated in one or more tissues (see above for rationale). Our approach is analogous to the one used by Zhang *et al*. [16], who observed that, in mice, OR gene expression is lowest in the vomeronasal organ (VNO), then used the expression levels in this tissue as a background against which to compare data from each of the other tissues. Since humans lack a clear VNO [32], and we could not find a tissue in which OR gene expression is clearly lower (Additional data file 1), we looked for elevated expression in each individual tissue compared to the distribution of expression levels across all the other tissues (including the olfactory epithelium). Using our approach, only 33 OR genes (Figure 2a; Additional data file 1) were found to have significantly elevated expression in non-olfactory tissues compared to olfactory epithelium (at an adjusted *P *< 0.05; corrected for multiple testing in five tissues). An obvious limitation of the approach is that if a gene is expressed at similar levels in all tissues, we would not be able to identify it as differentially expressed and hence would not detect it as expressed. Reassuringly, however, although our criteria for detection is somewhat different from that of Zhang *et al*., our finding is consistent with their results in mice in terms of the proportion of OR genes whose expression is enriched in the studied non-olfactory tissues [16].

When we compared expression among the non-olfactory tissues alone (that is, excluding the olfactory epithelium samples), we found 147 OR genes to have significantly elevated expression in one or more tissues (Figure 2b; Additional data file 1). We therefore considered the expression of these genes to be detected in those non-olfactory tissues. When we used RT-PCR on a sample of four genes in four tissues to validate our results, we found only one instance in which we did not confirm the findings of the array (Additional data file 4). Since we expect 29/578 genes to be false positives (given the statistical cutoff we used; *P *< 0.05), an error rate of 1/22 assays (4.5%) in the RT-PCR results for olfactory epithelium and non-olfactory tissues is consistent with our expectation. The RT-PCR also reveals expression in one case not detected using the array (for *OR2T1 *in kidney). Again, this false negative is expected given the increased sensitivity of RT-PCR, and the conservative criteria that we used to detect expression from the microarray data.

We note that 32 of the ectopically expressed OR genes (identified by comparison only to other non-olfactory tissues) were not identified as expressed in olfactory epithelium. This finding raises a question as to the functional annotation of these 32 genes as odorant receptors. If these genes are only expressed in non-olfactory tissues, they are unlikely to participate in odorant recognition. Interestingly, while the only existing hypothesis regarding additional functions of OR genes is about a possible role in sperm chemotaxis [15], we found that the tissues with the largest number of ectopically expressed OR genes were actually the lung and heart (Table 2). These results are in agreement with previous observations of ectopic expression of human OR genes based on ESTs and genome-wide expression microarrays [14]. In addition, although the approach used in [14] to analyze the microarray data is different than the one we used, the overlap in the lists of ectopically expressed OR genes in the two studies is significantly larger than expected by chance (*P *= 0.012; Additional data file 2).

**Table 2:** Number of expressed OR genes in non-olfactory epithelium tissues

	Liver	Heart	Kidney	Testis	Lung
Enriched compared to non OE*	14	44	13	21	56
Enriched compared to OE^†^	5	10	8	4	10

*Detected as expressed in each tissue based on elevated expression relative to all the other non-olfactory epithelium samples (at an adjusted *P *< 0.05). ^†^Detected as expressed in each tissue based on elevated expression relative to the olfactory epithelium samples (at an adjusted *P *< 0.05)

### Do OR genes have additional functions?

Since the first observation of ectopic expression of OR genes [12,13], it was hypothesized that odorant receptors may have additional functions in non-olfactory tissues. We performed evolutionary analysis of ectopically expressed OR genes in order to test this hypothesis. Our basis is the recent observation that genes expressed in a larger number of tissues evolve under stronger evolutionary constraint compared with genes that are expressed in one or a small number of tissues [33,34]. This observation was interpreted to reflect the greater number of evolutionary constraints imposed by the need to optimize function in multiple tissues relative to the functional constraints of expression in a single tissue.

By using a comparison of human-chimpanzee-rhesus orthologous OR genes (see Materials and methods), we found that human OR genes that are also ectopically expressed do not evolve under greater evolutionary constraint on the human lineage (median Dn/Ds on the human lineage = 0.62) than OR genes expressed exclusively in the olfactory epithelium (median Dn/Ds = 0.64; Mann-Whitney U; one tailed *P *= 0.60). This observation suggests that ectopic expression of human OR genes may not impose additional functional constraints on the odorant receptor protein. In addition, no significant differences in Dn/Ds ratios were found when we used protein domains (for example, specific trans-membrane domains or the putative binding sites [35]), rather than the entire coding region in our comparison (*P *> 0.32 for all comparisons). We note, however, that the individual protein domains are short (17-45 residues), and hence did not accumulate many substitutions along the human lineage; as a result, the analysis of individual protein domains may be underpowered to identify differences in selective pressures.

When we focused on homologous human and mouse OR genes (we use the term 'homologs' instead of 'orthologs' because determining orthology between human and mouse gene families is not straight-forward; see Materials and methods for details), we further found that they are no more likely to be ectopically expressed in the same tissue than expected by chance. Specifically, we could not reject the null hypothesis that homologous OR genes are expressed in heart, lung and testis (the tissues included both in our study and in [16]) at random (*P *= 0.55, *P *= 0.06, and *P *= 0.64 for a comparison of OR genes expressed in heart, lung, and testis, respectively, using a hyper-geometric distribution). Together, these observations suggest that the ectopic expression profiles of individual OR genes are not conserved across mammalian species. In summary, although we cannot exclude the possibility that a subset of OR genes have additional functions, overall, our results point to the random expression of a large number of mammalian OR genes in non-olfactory tissues with no functional significance, possibly due to leaky promoters. Thus, our results are consistent with the neutral explanation for ectopic expression of OR genes proposed by [14]. The comparison of ectopic OR gene expression variation within and between species would help to test this hypothesis more directly (reviewed in [36]).

## Conclusion

We detected the expression of 437 predicted human OR genes in olfactory epithelium, in support of their functional annotation as odorant receptors. In contrast, at least 32 predicted human OR genes may not be odorant receptors, as they appear to be expressed exclusively in non-olfactory tissues. A caveat is that, given the observed inter-individual variability in OR gene expression, a subset of these 32 OR genes may be expressed in other individuals. We also described abundant ectopic expression of human OR genes. However, our evolutionary analysis of ectopically expressed OR genes does not lend support to the hypothesis that odorant receptors have additional functions.

## Materials and methods

### The human OR gene microarray

We designed a custom Affymetrix human OR gene microarray with 1,561 probe-sets for 578 predicted human OR genes. Each probe-set contains 11 perfect match probes of 25-mers each, and 11 mismatch probes (in which a mismatch nucleotide is introduced at the center of the probe). This design is the same as for the commercially available Affymetrix genome-wide expression microarrays.

Since many OR genes are similar to each other at the coding region level, cross hybridization may be an issue. In order to avoid this problem, the expression of each human OR gene is measured by an average of 2.7 probe-sets (range 1-10), with at least one probe-set designed in predicted 3' untranslated regions of the OR genes. Given their level of similarity, the untranslated regions of OR genes are not expected to be affected by cross-hybridization more than any random probe-set on an Affymetrix array (Additional data file 5). In addition, we excluded from our analysis probes that Affymetrix identified as more likely to be susceptible to cross-hybridization, based on a whole genome search (this procedure did not result in the exclusion of OR genes from the analysis).

The 578 OR genes that are represented on the array include 366 of the 379 (97%) predicted human OR genes with intact full length (>270 residues) coding regions. The remaining 212 probe-sets were designed for human OR pseudogenes. Of the 212, eight are short sequences (less than 270 residues) that contain no stop codon, and 120 contain only one stop codon in the first 270 residues or more. If the mutation that causes the stop codon is segregating in human populations (as is the case for 26 of these single disruption OR pseudogenes [30]), annotated pseudogenes may in fact be functional odorant receptors in some individuals [30,37].

In addition to probes for OR genes, the microarray contains probe-sets for 33 'house keeping' genes that can be used as controls for hybridization quality, and for the normalization of the arrays (Additional data file 6).

### Hybridization and pre-processing of the data

Hybridizations and scanning of the arrays were performed at the University of Chicago Functional Genomics Facility using an Affymetrix GeneArray Scanner 3000. Since OR genes are expected to be expressed mainly in olfactory epithelium, the overall intensity of hybridizations with RNA from olfactory epithelium is expected to be higher than the overall intensity of hybridizations of RNA from other tissues. As a result, standard normalization methods [38,39] would have the effect of artificially inflating the estimates of the non-olfactory epithelium expression levels to those seen in olfactory epithelium [16]. Instead, we proceeded by performing a quantile normalization on the raw intensity values of either the olfactory epithelium or the non-olfactory epithelium tissues separately, followed by an experiment-wide normalization, based only on the Affymetrix control probe-sets and 20 probe-sets for non-OR genes (Additional data file 6). We then used the robust multi-array average (RMA) algorithm [38] to obtain one expression estimate for each probe-set (see Additional data file 6 for more details). RMA values for all human OR gene probe-sets are available in Additional data file 3.

### Statistical analysis

We fit the following linear mixed effects model:

*R*_*ijk *_= *α*_*i *_+ *β*_*j *_+ ε_*ijk*_

where we have suppressed the probe-set labels. Here, *R*_*ijk *_is the normalized log transformed RMA value (of a particular probe-set) for technical replicate *k *of a particular tissue sample *j*; the label *i *is used to indicate the tissue(s) used in the comparison (for example, olfactory epithelia compared to non-olfactory tissues). The term *β *is a random effect for the tissue sample *j*, assumed to be uncorrelated with mean zero and variance σβ2
 MathType@MTEF@5@5@+=feaafiart1ev1aaatCvAUfeBSjuyZL2yd9gzLbvyNv2Caerbhv2BYDwAHbqedmvETj2BSbqee0evGueE0jxyaibaiKI8=vI8tuQ8FMI8Gi=hEeeu0xXdbba9frFj0=OqFfea0dXdd9vqai=hGuQ8kuc9pgc9s8qqaq=dirpe0xb9q8qiLsFr0=vr0=vr0dc8meaabaqaciGacaGaaeqabaqadeqadaaakeaacqaHdpWCdaqhaaWcbaGaeqOSdigabaGaaGOmaaaaaaa@377A@. The term *ε*_*ijk *_is the residual error term (technical variance), and is assumed to be uncorrelated with mean zero and variance σε2
 MathType@MTEF@5@5@+=feaafiart1ev1aaatCvAUfeBSjuyZL2yd9gzLbvyNv2Caerbhv2BYDwAHbqedmvETj2BSbqee0evGueE0jxyaibaiKI8=vI8tuQ8FMI8Gi=hEeeu0xXdbba9frFj0=OqFfea0dXdd9vqai=hGuQ8kuc9pgc9s8qqaq=dirpe0xb9q8qiLsFr0=vr0=vr0dc8meaabaqaciGacaGaaeqabaqadeqadaaakeaacqaHdpWCdaqhaaWcbaGaeqyTdugabaGaaGOmaaaaaaa@3780@. We used this model to estimate whether the difference in gene expression, *α *_1 _- *α *_2_, between olfactory epithelium and non-olfactory tissues is significantly greater than zero (using a one-tailed *t*-test). We used the same procedure to compare gene expression only among the non-olfactory tissues (see Additional data file 6 for more details).

### Analysis of OR gene orthologs

To identify human-chimpanzee-rhesus ortholog trios, we first obtained the collection of rhesus OR genes. To do so, we used 117 representative OR protein sequences from human and mouse [35] in tblastn [40] searches against the entire rhesus genome sequence (downloaded from the human genome sequencing center at Baylor college of medicine [41] on February 17th 2006), and collected all results with an E-value cutoff of 10^-4^. We then merged overlapping results and obtained a set of 756 putative rhesus OR gene sequences which were at least 300 bp long (YG and Orna Man, unpublished results).

Human-rhesus reciprocal best hits were obtained by using blastx [42] searches of each of the 756 rhesus OR gene sequences (since a reliable translation of the rhesus sequences could only be obtained at a later stage - see below) against the human OR protein sequences (obtained from build 41 of the HORDE database [43]), and by using tblastn for the reciprocal searches. Human-chimpanzee reciprocal best hits were obtained using two-way blastp [44] searches of the two protein collections [45]. Finally, 360 human-chimpanzee-rhesus clear ortholog trios were determined by merging human-chimpanzee and human-rhesus ortholog pairs with a common human gene. The nucleotide sequences of each trio were aligned using clustalW [46] with default parameters, and the human protein sequence was used to create an in-frame alignment that excludes stop codons and insertions/deletions in the other species [45]. Using the ortholog sequences of the three species, lineage-specific Dn/Ds ratios were estimated using the codeml program from the PAML package [47], with model number 1 (allowing a separate Dn/Ds value for each lineage).

In contrast to the result for primates, only 218 human-mouse clear orthologs [35] could be identified by using the reciprocal best hit approach (because of the many gene duplications and deletions since the human-mouse common ancestor). Of the 218, only 33 were shown to have ectopic expression in mice [16]. This number is too small for an analysis of shared expression profiles across species (see results). Instead, for each of the mouse OR genes that were ectopically expressed in [16], we identified the human OR gene with the highest sequence similarity. While this analysis does not yield clear orthologs, it reveals the most similar sets of human-mouse homologous OR genes. In the absence of reciprocal best hits, the same human gene might be assigned as the homolog of more than one mouse OR gene. The consequence is that we are less likely to observe common expression profiles between mouse and human genes than if we could obtain a list of true orthologs.

### Electronic database information

All expression data and original CEL files were submitted to the GEO database under the series accession number [GSE5969].

## Additional data files

The following additional data are available with the online version of this paper. Additional data file 1 is a table of *P *values for all OR genes in all tissues. Additional data file 2 includes the calculation for the overlap of ectopically expressed genes in our study and that of Feldmesser *et al*. Additional data file 3 is a table of RMA values (in log scale) for all probe sets from all hybridizations. Additional table 4 is a figure of the RT-PCR validation of the microarray results. Additional data file 5 is a figure of the analysis of co-similarity of either the untranslated or the coding region probe-sets. Additional data file 6 provides supplementary materials and methods.

## Supplementary Material

Additional data file 1 *P *values for all OR genes in all tissues. Click here for file

Additional data file 2 Calculation for the overlap of ectopically expressed genes in our study and that of Feldmesser *et al*. [14]. Click here for file

Additional data file 3 RMA values (in log scale) for all probe sets from all hybridizations. Click here for file

Additional data file 4 RT-PCR validation of the microarray results. Click here for file

Additional data file 5 Analysis of co-similarity of either the untranslated or the coding region probe-sets. Click here for file

Additional data file 6 Supplementary materials and methods. Click here for file
