# Supplementary material for: Characterizing the expression of the human olfactory receptor gene family using a novel DNA microarray
Source: Genome Biol. 2007 May 17;8(5):R86. doi: 10.1186/gb-2007-8-5-r86 (PMC1929152; doi:10.1186/gb-2007-8-5-r86)
Supplement: Additional data file 4 — RT-PCR validation of the microarray results. [file gb-2007-8-5-r86-S4.doc]

**Validation using RT-PCR.**

In order to validate the results of the microarray analysis, we chose at random 10 OR genes, six of which were detected as expressed only in olfactory epithelium, and four genes of which were detected as expressed both in olfactory epithelium and in at least one non-olfactory tissue. We designed primers that amplify the coding regions of these 10 genes (primer sequences available upon request), and performed RT-PCR using as template the same RNA that was used for the array hybridization for the relevant tissues (i.e., olfactory epithelium, liver, kidney, and heart; the tissues in which expression of these 10 genes was detected). RNA from the olfactory epithelium samples was pooled.

As can be seen in figure 1 of the paper, RNA from these tissues does not contain DNA contamination. Shown here are gel electrophoresis pictures of the RT-PCR products, as well as a table that summarizes the results. As can be seen, we confirmed the expression of all 10 OR genes in olfactory epithelium. The expression of only one gene (*OR2T1*) could not be confirmed in a non-olfactory tissue (heart). This result is consistent with the statistical cutoff we used to detect OR gene expression (see main text).

Using RT-PCR, we also amplified the product for the gene *OR2T1* in kidney, although this gene was not detected as expressed in the microarray data. This is not a surprising result, as RT-PCR amplification is expected to be more sensitive than microarray hybridization.


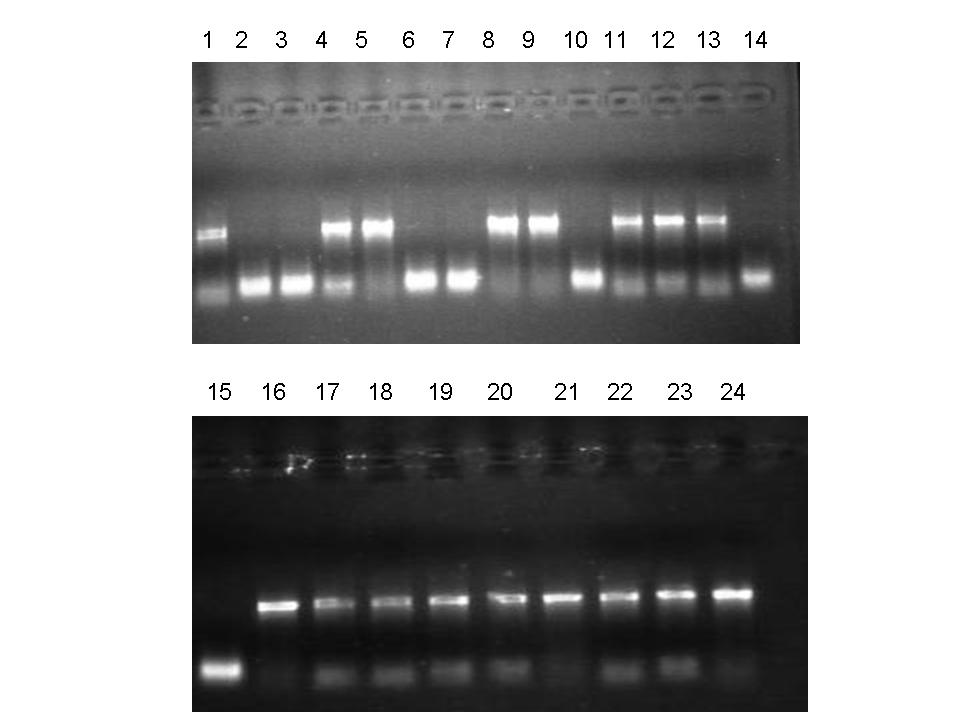


The RT-PCR amplifications by lane (primer pair / template): lane #1: OR2T1/OE; #2: OR2T1/heart; #3: OR2T1/liver; #4: OR2T1/kidney; lane #5: OR6N2/OE; #6: OR6N2/heart; #7: OR6N2/liver; #8: OR6N2/kidney; lane #9: OR10G4/OE; #10: OR10G4/kidney; #11: OR10G4/liver; #12: OR10G4/heart; lane #13: OR6N2/OE; #14: OR6N2/heart; #15: OR6N2/liver; #16: OR6N2/kidney; #17: OR7D4/OE; #18: OR5K1/OE; #19: OR8J3/OE; #20: OR6S1/OE; #21: OR5B1P/OE; #22: OR3A4/OE;#23: OR3A4/positive control using genomic DNA; #24: OR6S1/positive control using genomic DNA

The RT-PCR results are summarized in the table below. (+) indicates a successful amplification; (-) indicates a negative result. The shaded cells in the table are those in which, based on the results from the array, we expect to successfully amplify a product.

| Gene/Tissue | Olfactory epithelium | Heart | Liver | Kidney |
| --- | --- | --- | --- | --- |
| *OR2T1* | **+** | **-** | **-** | **+** |
| *OR10G4* | **+** | **+** | **+** | **-** |
| *OR2L1P* | **+** | **-** | **-** | **+** |
| *OR6N2* | **+** | **-** | **-** | **+** |
| *OR7D4* | **+** |  | | |
| *OR5K1* | **+** |  | | |
| *OR8J3* | **+** |  | | |
| *OR6S1* | **+** |  | | |
| *OR5B1P* | **+** |  | | |
| *OR3A4* | **+** |  | | |
