# Supplementary material for: Characterizing the expression of the human olfactory receptor gene family using a novel DNA microarray
Source: Genome Biol. 2007 May 17;8(5):R86. doi: 10.1186/gb-2007-8-5-r86 (PMC1929152; doi:10.1186/gb-2007-8-5-r86)

Presented is an analysis of co-similarity of either the untranslated (UTR) or the coding region probe-sets. As can be seen, less than 13% of the coding region probe-sets that we include in the analysis are more than 70% similar (all those that are 100% identical were excluded based on the information provided by Affymetrix). In addition, only a few (~2%) of the UTR probe-sets that we included in the analysis are more than 70% similar at the nucleotide level. Hence, cross hybridization is not expected to affect the UTR probe-sets more than it affects arrays for non-gene families.


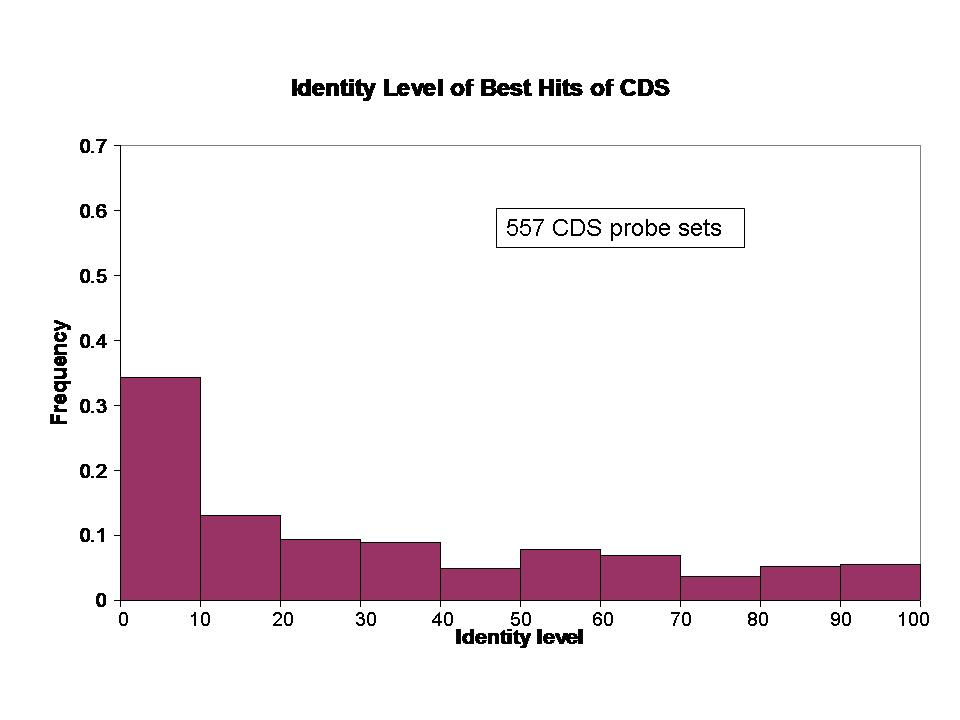


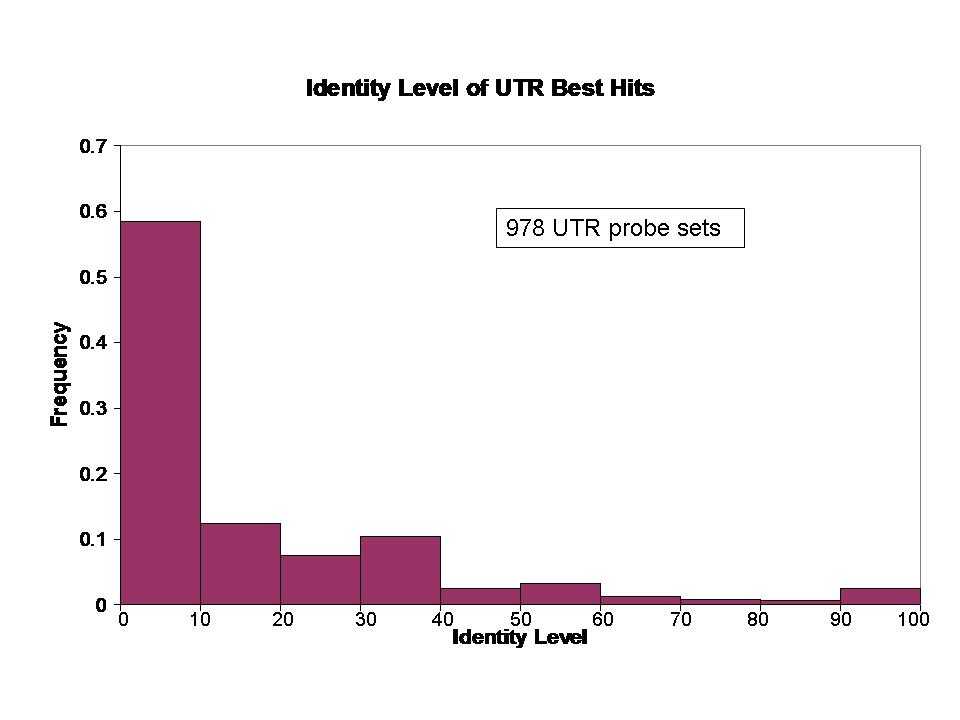

Supplement: Additional data file 5 — Analysis of co-similarity of either the untranslated or the coding region probe-sets. [file gb-2007-8-5-r86-S5.doc]
