# Supplementary material for: Characterizing the expression of the human olfactory receptor gene family using a novel DNA microarray
Source: Genome Biol. 2007 May 17;8(5):R86. doi: 10.1186/gb-2007-8-5-r86 (PMC1929152; doi:10.1186/gb-2007-8-5-r86)
Supplement: Additional data file 6 — Supplementary materials and methods. [file gb-2007-8-5-r86-S6.pdf]

Supplementary Methods

The analysis of the data used elements from the RMA pipeline proposed by Irizarry *et al.*[49][50]. We also used some of the routines contained in the package `affy` [51], which is part of the Bioconductor project [52], and is the standard implementation of the RMA approach. The raw data from which we started our analysis were the probe level intensity measures.

Background correction. We attempted to correct for background using three different procedures (optical correction only, correction based on probe composition only, or both), using routines from the package `gcrma`.

OPTICAL BACKGROUND CORRECTION. The model assumes that the raw intensity data is the result of the sum of a true signal, which is assumed to follow an exponential distribution, and optical noise, which is assumed to be normally distributed. The corrected expression value is obtained as the expected value of the signal component conditional on the observed value. [50].

BACKGROUND CORRECTION BASED ON PROBE COMPOSITION. This model (implemented in `gcrma`) uses the sequence information (base composition and position of each nucleotide) of each probe (obtained from the CDF file corresponding to the array) to predict and correct for non-specific binding affinity.

As can be seen in Figure 1, the correlation between the log transformed PM values of the two technical replicates for each tissue tends to decrease after applying any of the background correction procedures. This indicates that the proportion of "technical noise" (noise not attributable to biological variability) is amplified by these background correction procedures [53]. We concluded that background correction should not be performed in this case, as it reduces the true signal.

As a result, the data used in all subsequent analyses was not corrected for background and was only transformed by taking base 2 logs.

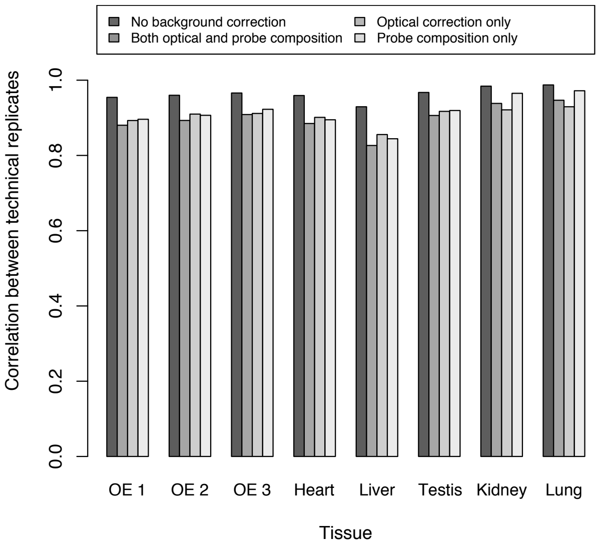

Figure 1: *Correlations between the* $\log_2(PM)$ *values for the two technical replicates corresponding to each sample. The color indicates the kind of background correction performed before computing the correlation.*

Normalization. Normalization of microarray data is critical for removing systematic non-biological effects in any expression study to allow for a comparison across arrays. For the normalization and computation of the RMA summaries, we use only the PM

(perfect match) intensities from each probe pair, and used the method of *quantile normalization*, as suggested by Bolstad *et al.* [54]. The computations were performed using some of the normalization routines in the `affy` package.

Since OR genes are expected to be expressed mainly in olfactory epithelium, the overall intensity of hybridizations with RNA from olfactory epithelium is expected to be higher than the overall intensity of hybridizations of RNA from other tissues. As a result, standard normalization methods would have the effect of artificially inflating the estimates of the non-olfactory epithelium expression levels. To avoid that, we split the data in two groups, corresponding to OE samples and non-OE samples, respectively, and applied the quantile normalization method to each group separately. As a second step, the distributions for the two groups (OE's and non-OE's) were adjusted based on the level of expression of a set of housekeeping genes (see below).

Computation of the robust multi-array average (RMA) values.

After the PM intensities have been normalized (separately for OE and non-OE tissues), the RMA expression index [49] was obtained by fitting the model

$$\log_2\left(PM_{ij}^{(k)}\right) = \alpha_i^{(k)} + \beta_j^{(k)} + \epsilon_{ij}^{(k)},$$

where i ranges over the probes, k over the probe-sets, j over the arrays, and $\epsilon_{ij}^{(k)}$ is a stochastic error term. This model is fitted using Tukey's robust *medianpolish* method. The resulting $\hat{\beta}_j^{(k)}$ is the expression value for probe-set k in array j. This procedure was performed using routines from the package `affy`. The resulting expression indices are in the $\log_2$ scale.

Notice that in fitting this model we do not need (and should not) separate the different tissues, since the systematic differences become part of the fitted $\hat{\beta}$'s.

Adjustment based on housekeeping genes. To adjust the distributions of the two groups of arrays (OE and non-OE samples), we use a set of 33 housekeeping genes (20 included in the arrays by our design, and 13 added by Affymetrix), whose expression is expected to remain close to constant through the samples (we checked this assumption; see below). This step is not part of the standard RMA pipeline.

We computed an additive offset term by fitting the following linear model:

$$X_{ijk} = \alpha_i + \beta_j + \epsilon_{ijk},$$

where X_{ijk} is the RMA value; i equals 1 or 2, depending on whether the sample comes from OE or not; j ranges over the housekeeping genes; and k identifies the specific array. The error terms ϵ_{ijk} are assumed to be independent and normal, with variance σ_j^2 that depends on the gene.

To fit the model above, we used an iterative reweighting procedure: at each step, we used weighted least squares to fit the model above, using as weights $\{\rho_j\}$ the sample variances of the residuals grouped by gene j from the previous step. This procedure converged in about 10 iterations. However, during this procedure, we found that 3 of the Affymetrix housekeeping genes produced a few extreme outliers (i.e., their expression cannot be assumed to be constant across all samples); these genes were dropped altogether from the procedure (leaving a total of 30 housekeeping genes).

Since we set $\alpha_1 = 0$, the term $\hat{\alpha}_2$ is used as the offset to be subtracted from the RMA values for the non-OE samples. The fitted value $\hat{\alpha}_1$ we estimated was 0.334. See Figure 2 for a residual plot of the final least squares fit.

Tests of differential expression. These tests were performed probe-set by probe-set, using mixed-effects linear models. The small number of degrees of freedom is a concern, but it has been argued (see for example, Cui and Churchill [55], in the context of cDNA arrays) that the method is appropriate. We visually checked residual plots for fits corresponding to several different probe-sets and found no reasons to think that the distributional assumptions needed are violated.

Testing for differential expression in the OE and non-OE samples. To detect differentially expressed genes we consider the following mixed-effects model:

$$R_{ijk} = \alpha_i + \beta_j + \epsilon_{ijk},$$

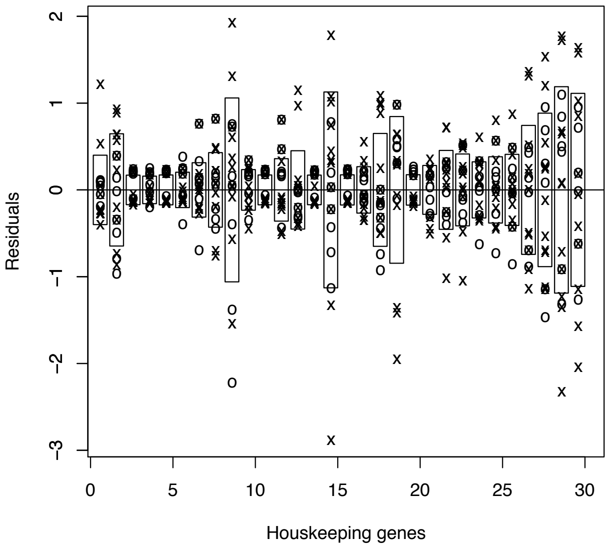

Figure 2: *Residual plot for the computation of the adjustment based on housekeeping genes. "o" denotes data points from OE samples, while "x" denotes points from non-OE samples. The bars indicate the size of the corresponding weights, as obtained at the end of the iterative re-weighting procedure (the length of each bar is twice the square root of the reciprocal of the final weights).*

where $i = 1, 2$ indicating whether the tissue is OE or not, j ranges over the tissue sample, and k identifies the technical replicate; here we consider the tissue effect to be random. That is, β represents the biological variability, while ϵ represents the technical variability. In order to obtain a p-value for each probe-set, we assume that the β_j's are i.i.d. $N(0, \sigma_\beta^2)$ and the ϵ_{ij}'s are i.i.d. $N(0, \sigma_\epsilon^2)$.

Setting $\alpha_1 = 0$, we performed tests of the null hypothesis that $\alpha_2 = 0$ versus one-sided alternatives; these tests were also repeated by selecting only some of the OE samples or some of the non-OE samples. The fitting of these mixed models and the testing was performed using the R package `nlme`.

Testing for differential expression among the non-OE samples. For each of the non-OE tissues, we tested each probe-set for higher expression over the other non-OE tissues. We used the same setup as above, now with i indicating whether the array corresponds to the selected tissue or not. In these tests the OE samples were not used.

Combining p-values from different probe-sets. Each of the tests above provided a p-value per probe-set. Since in our array design many genes have more than one probe-set, we combined the p-values from different probe-sets to obtain a single p-value per gene per test by using Fisher's combination: the sum of 2 times the natural log of the p-values has, under the null hypothesis, a chi-square distribution with N degrees of freedom, and we can used that to obtain a combined p-value (see, for example, [56]).
